# Case Report: Rosai-Dorfman Disease Involving Sellar Region in a Pediatric Patient: A Case Report and Systematic Review of Literature

**DOI:** 10.3389/fmed.2020.613756

**Published:** 2020-11-30

**Authors:** Yi Zhang, Jie Liu, Jianyu Zhu, Xiang Zhou, Kun Zhang, Shirui Wang, Wenbin Ma, Hui Pan, Renzhi Wang, Huijuan Zhu, Yong Yao

**Affiliations:** ^1^Department of Neurosurgery, Peking Union Medical College, Peking Union Medical College Hospital, Chinese Academy of Medical Science, Beijing, China; ^2^Department of Endocrinology, Peking Union Medical College, Peking Union Medical College Hospital, Chinese Academy of Medical Science, Beijing, China

**Keywords:** Rosai-Dorfman disease, sellar region, treatment strategies, MRI manifestation, clinical presentation

## Abstract

Rosai-Dorfman disease (RDD) is an extremely rare histiocytic disorder characterized by cervical lymphadenopathy, while the involvement of sellar region is less observed. Here we report a pediatric patient who was initially suspected as sellar germinoma but later identified as RDD. We also conducted a systematic review about RDD involving sellar region. A total of only 14 cases were included and analyzed in our study in terms of clinical presentation, endocrine abnormality, radiological features, pathology, treatment, and follow up. The most common neurological manifestations of sellar RDD is diabetes insipidus and visual changes. Two typical kinds of MRI manifestations were presented in sellar RDD; one is like meningioma-like mass lesions, another showing infiltrative pattern that demonstrates hyperintense areas on T2WI. Currently, the treatment of RDD is tailored to the individual clinical circumstances. For sellar RDD, surgical treatment can be considered to completely remove or debulk the tumor.

## Introduction

Rosai-Dorfman disease was first reported in 1969 as sinus histiocytosis with massive lymphadenopathy, which was a benign and progressive lymphoproliferative disorder presenting with lymphadenopathies and polyclonal hypergammaglobulinemia (1, 2). RDD is more frequently seen in children and young adults than in older adults (3), while it seems not to present a explicit sex predilection (4). In addition, extranodal involvement has been reported in 43% of RDD, with the most common sites being the skin and the central nervous system (CNS) (5, 6). CNS involvement occurs in <5% of cases with 75% occurring as intracranial and 25% as spinal lesions (5). Patients with primary CNS involvement are often free of the general symptoms mentioned above and may manifest with only neurological symptoms related to lesion location such as headache, seizure, and focal neurological deficits (7).

Typical manifestations of RDD include non-specific painless lymphadenopathy (most often cervical and less commonly retroperitoneal, mediastinal, axillary, or inguinal) accompanied by fever, an elevated erythrocyte sedimentation rate, elevated inflammatory markers, mild anemia, and weight loss (2, 6). Other common symptoms include subcutaneous masses, bone pain, constitutional symptoms, and abdominal pain (8). Surprisingly, extra nodal presentations were more common than nodal lesions (4, 8).

Up to 70% of patients show no associated lymphadenopathy. The most common site of CNS involvement is the dura as well as the parasellar, cavernous sinus, petroclival, and parafalcine regions—the cerebellopontine angle and posterior fossa (9), but the pituitary involvement of RDD is extremely rare. Although some authors reported some cases, none of them have summarized these comprehensive data in a systematic way. Here, we report a case of pediatric RDD with primary pituitary involvement. And a systematic review of 14 cases of symptomatic sellar RDD is conducted (Tables 1, 2).

**Table 1:** Clinical summary of 14 RDD patients involved in sellar region in terms of clinical presentation and endocrine abnormality.

**Authors and year**	**Ref.no**	**Gender/age**	**Clinical presentation**			**Clinical course**	**Physical examination**			**Endocrine abnormality**	
			**Symptoms**	**Fever**	**Diabetes insipidus**		**Palpable lymphadenopathy (location)**	**Visual acuity and field**	**Others**	**Pre-op endocrine examination**	**Post-op endocrine examination**
Bhattacharjee et al., 1992	(10)	M 78	Bilateral visual impairment, mild headache, visual blurring	–	–	8 m		Bitemporal hemianopia, right optic atrophy, and right relative afferent pupillary defect	None	NA	NA
Ng et al., 1995	(11)	M 22	Polydipsia, polyuria, lack of libido, decreased frequency of shaving	–	+	6 m	–	NA	Obese, lack secondary sexual characteristics	Elevated prolactin, low testosterone	NA
Kelly et al., 1999	(12)	F 45	Headaches, pyrexia, vomiting, bilateral discharge from ears, amenorrhea, weakness and numbness of both legs, unsteady gait, facial pain, nasal obstruction, polyuria, thirst	+	+	3 m	–	NA	Neck stiffness, otitis externa, joint position sense and vibration sense lost in both legs and pin prick and light touch diminish below T2, increased knee and ankle reflexes	Low cotisol, prolactin, TSH, LH, T4, E2, IGF-1	NA
Woodcock et al., 1999	(13)	F 15	Amenorrhea, headache, blurred vision	–	–	7 y	–	Blurred vision	Normal	NA	NA
Wan et al., 2008	(14)	M 43	Visual blurring of the left eye, headache	–	–	1 m	–	Vision impairment of the left eye and bilateral temporal visual field defect	NA	NA	NA
Rotondo et al., 2010	(15)	F 63	Ataxia, diarrhea, weight loss, abdominal pain	–	–	6 w	–	NA	NA	Low cortisol, thyroxine, gomadotrophins	NA
Gupta et al., 2011	(16)	M 14	Loss of vision in the left eye	–	–	1 y	–	Loss of vision in the left eye	NA	Elevated T4, prolactin and cortisol	NA
Wang et al., 2011	(17)	F 10	Polydipsia, polyuria	–	+	1 y	–	Normal	Length and weight were under 3.97-ft (121 cm) and 22-kg. Her hands and feet were smaller than normal for her age, rough skin	Low cortisol, thyroxine, growth hormone, and gonadotrophins	NA
Wang et al., 2011	(17)	M 27	Polydipsia, polyuria, headache, visual impairment, weakness, decreased libido	–	+	1 y	–	A reduction of visual acuity, bitemporal hemianopia	NA	NA	subnormal growth hormone, gonadotropin, and cortisol
Cangelosi et al., 2011	(18)	F 50	NA	–	NA	NA	–	NA	Multiple cutaneous papules, measuring up to 0.5 cm, on her bilateral axilla and medial thighs, just below her groin	NA	NA
Chandrashekhara et al., 2011	(19)	M 30	Headache, vomiting, diminished vision	–	–	2 y	cervical	Diminished vision	Multiple cervical lymphadenopathies, enlargement of submandibular glands	NA	NA
Sasidharan et al., 2020	(20)	F 32	Progressive weakness of the right arm, headache, diminution in bilateral eyes	–	–	2 y	neck nodes	Diminution in bilateral eyes	NA	NA	NA
Sasidharan et al., 2020	(20)	M 29	Progressive reduction of vision in the left eye	NA	NA	NA	–	Progressive reduction of vision in the left eye	NA	NA	NA
Our case		M 10	Polyuria and polydipsia	–	+	2 y	–	–	Chest discomfort, fatigue, hypohidrosis, hot flush of palms, and being afraid of heat	Low levels of LH, T and slightly high level of PRL	–

**Table 2:** Clinical summary of 14 RDD patients involved in sellar region in terms of radiological features, pathology, treatment, and follow up.

**Authors and year**	**Ref.no**	**Blood workup**				**Radiological features**				**Pathology**				**Solitary lesion or multifocal lesion (detail)**	**Treatment**	**Follow up**
		**Elevated neutrophile granulocyte**	**ESR**	**hyperglobulinemia**	**Anemia**	**Max mass size (cm)**	**MRI T1WI (isointense hypo- hyper-)**	**MRI T2WI (isointense hypo- hyper-)**	**Contrast enhancement (heterogeneous homo-)**	**S100**	**CD1a**	**CD68**	**Vimentin**			
Bhattacharjee et al., 1992	(10)	NA	Normal	NA	–	–	–	–	–	+	NA	NA	NA	Solitary lesion	Surgery	1 y alive
Ng et al., 1995	(11)	NA	NA	NA	NA	1.2 cm in diameter	NA	NA	Heterogeneous	NA	NA	NA	NA	Solitary lesion	Surgery	NA
Kelly et al., 1999	(12)	NA	55	NA	NA	2 cm in diameter	NA	NA	Heterogeneous	+	NA	NA	NA	Multifocal lesion	Surgery	3 y alive
Woodcock et al., 1999	(13)	NA	NA	NA	NA	NA	Isointense	Isointense	Heterogeneous	+	NA	NA	NA	Solitary lesion	Medication	9 m slight interval increase in the size of the lesion
Wan et al., 2008	(14)	Normal	Normal	–	–	3 × 3 × 2.5	Isointense	Isointense	Heterogeneous	+	–	+	NA	Solitary lesion	Surgery	3 m alive
Rotondo et al., 2010	(15)	NA	NA	NA	99–mild	1.7 × 0.7 × 0.2	NA	NA	NA	NA	NA	+	NA	Multifocal lesion	Surgery	Perioperative period died
Gupta et al., 2011	(16)	Normal	Elevated	–	–	NA	NA	Hypointense	Homogeneous	+	NA	NA	NA	Solitary lesion	Surgery	NA
Wang et al., 2011	(17)	Normal	Normal	–	–	NA	Hyperintense	NA	Heterogeneous	+	NA	+	NA	Multifocal lesion	Surgery	6 y alive
Wang et al., 2011	(17)	NA	elevated	NA	NA	NA	Isointense	Isointense	Heterogeneous	+	–	+	NA	Multifocal lesion	Surgery	5 y alive
Cangelosi et al., 2011	(18)	NA	NA	NA	NA	NA	NA	NA	NA	+	–	+	NA	Multifocal lesion	Surgery	NA
Chandrashekhara et al., 2011	(19)	NA	NA	NA	NA	NA	Isointense	Isointense	Heterogeneous	+	–	+	NA	Solitary lesion	Surgery	NA
Sasidharan et al., 2020	(20)	NA	NA	NA	NA	NA	NA	NA	Homogeneous	+	–	NA	NA	Multifocal lesion	Surgery+ Radiotherapy	Free of disease progression 35 months after therapy
Sasidharan et al., 2020	(20)	NA	NA	NA	NA	NA	NA	NA	Homogeneous	+	–	NA	NA	Solitary lesion	Surgery+ Radiotherapy	Alive with improvement in his vision after 25 months of therapy
Our case		A higher proportion of lymphocyte and a lower proportion of neutrocyte	Normal	–	–	NA	–	–	Homogenous	+	Sparesly positive	+	NA	Solitary lesion	Surgery+ Chemotherapy	Symptoms of polyuria and polydipsia persisted 2 ys later

## Case Presentation

### History and Examination

A 10-year-old boy presented to our clinic in September 2018 with polyuria and polydipsia without obvious precipitating factors for 2 months and chest discomfort for over half of a month. His urine output was about 8,000 ml per day. Urine and plasma osmotic pressure were 88 and 310 mOsm/kgH2O, respectively. Water deprivation test was positive. The patient also complained of fatigue, hypohidrosis, hot flush of palms and being afraid of heat. His previous history included cerebral hemorrhage and concussion due to traffic accident in the year of 2014. No sequelae were left.

**Figure 1:**
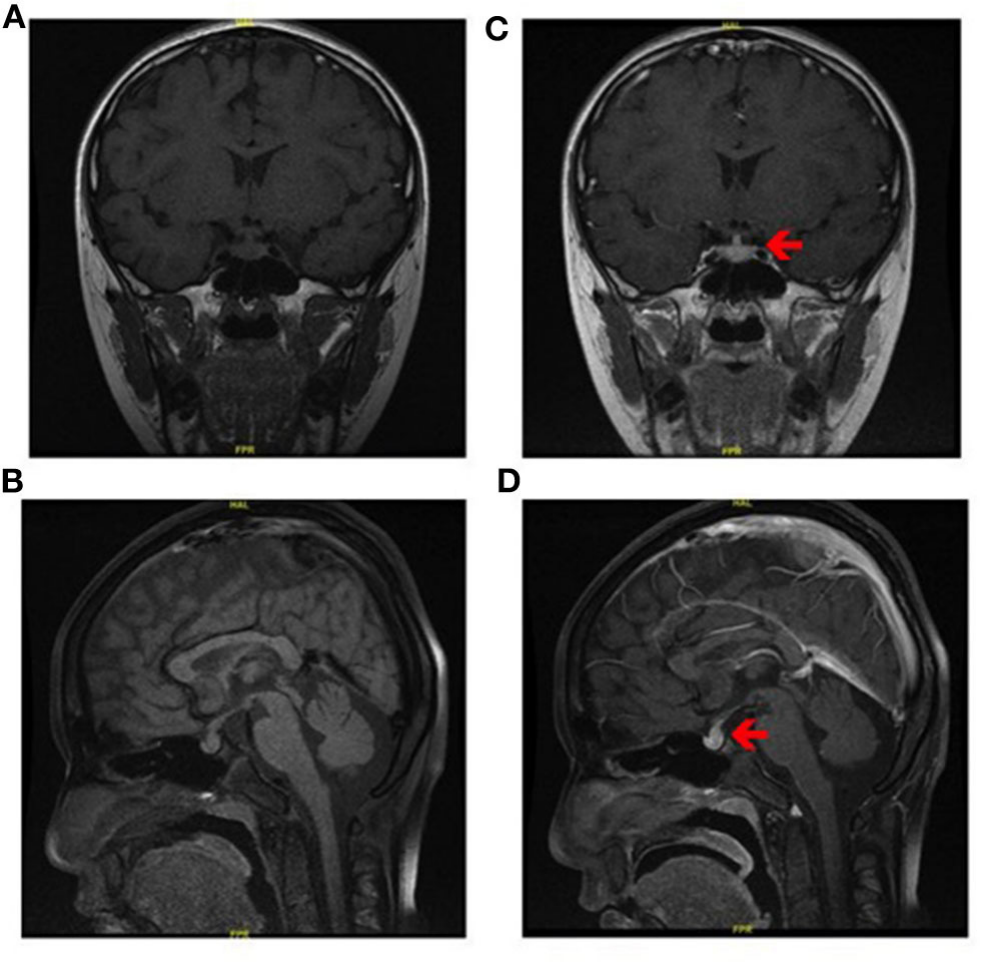
MRI characteristics of Rosai-Dorfman disease involving sellar region. **(A)** Coronal and **(B)** sagittal T1-weighted magnetic resonance images. **(C)** coronal and **(D)** sagittal contrast-enhanced magnetic resonance images reveal thickened pituitary stalk with obvious homogenous enhancement (red arrow).

Clinical examination was normal except for slight subxiphoid tenderness. Neither lymphadenopathy nor hepatosplenomegaly was observed. Contrast-enhanced brain MRI showed that the pituitary stalk was thickened with obvious homogenous enhancement and the high signal of posterior pituitary was disappeared in T1-weighted images (Figure 1). Examination of CSF revealed normal β-hCG (0.69 IU/L, normal range: 0–5 IU/L) and AFP (<0.605 ng/ml), normal glycorrhachia, protein, and chlorine levels, normal cell count. Laboratory test showed a higher proportion of lymphocyte (41.4%, normal range: 20–40%) and a lower proportion of neutrocyte (49.6%, normal range: 50–75%). Endocrine studies revealed low levels of LH (0.98 IU/L, normal range: 1.24–8.62 IU/L), T (0.14 ng/ml, normal range: 1.75–7.81 ng/ml), and slightly high level of PRL (16.54 ng/ml, normal range: 2.64–13.13 ng/ml). Plasma AFP and β-hCG was normal (3.7 ng/ml and 0.79 IU/L, respectively).

Considering his medical history, radiological test, examination of CSF and plasma, a diagnosis of central diabetes insipidus was initiated and possibly the germinoma, which required surgical biopsy to confirm it.

### Surgical Biopsy and Histological Findings

**Figure 2:**
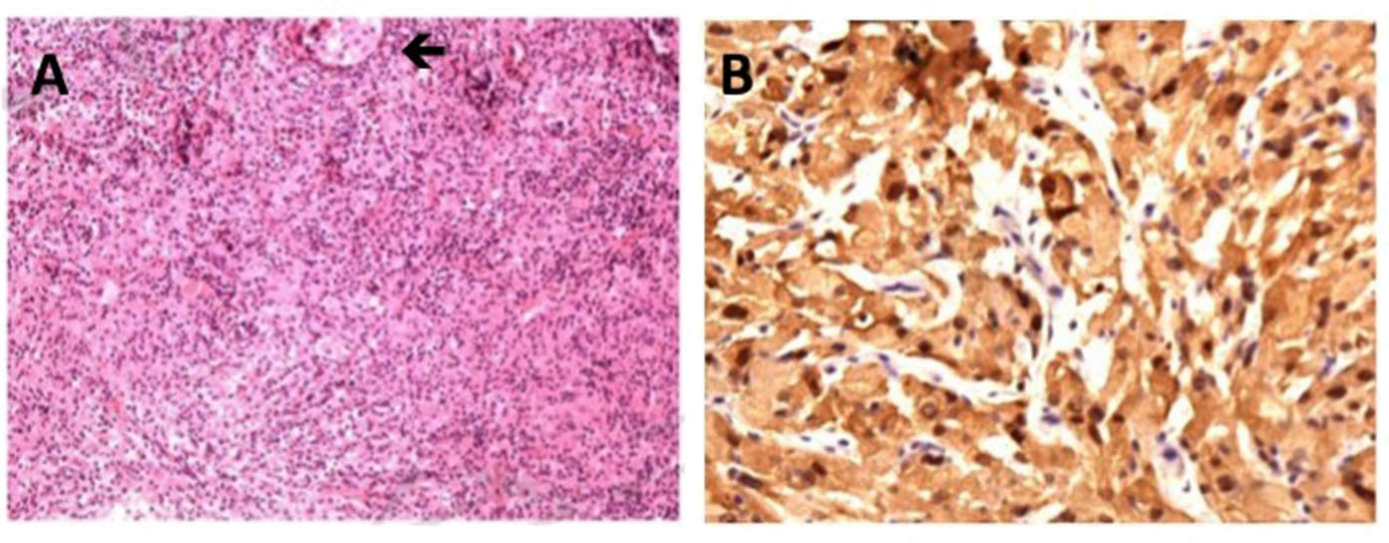
Histological features of Rosai-Dorfman disease involving sellar region. **(A)** H-E staining of specimen reveals the large-size histiocyte with the typically foamy eosinophilic cytoplasm, original magnification ×40 (black arrow). **(B)** Positive immunohistochemical staining for S-100, original magnification ×100.

Two days after admission, the patient underwent surgical biopsy using endoscopic transsphenoidal approach. The mass tissue was in the posterior sella with soft texture, gray-white color, and insufficient blood supply. H-E staining of specimen revealed the large-size histiocyte with the typically foamy eosinophilic cytoplasm. Immunohistochemical study revealed the histiocytes were positive for CD68, S-100, and negative for Langerin. The index of CD1a and CD21 were sparsely positive in the tissue. Only a few cells expressed CD3 and CD20. Cell proliferation index Ki-67 was 5%. The diagnosis of Rosai-Dorfman disease was therefore yielded (Figure 2).

### Postoperative Course

**Figure 3:**
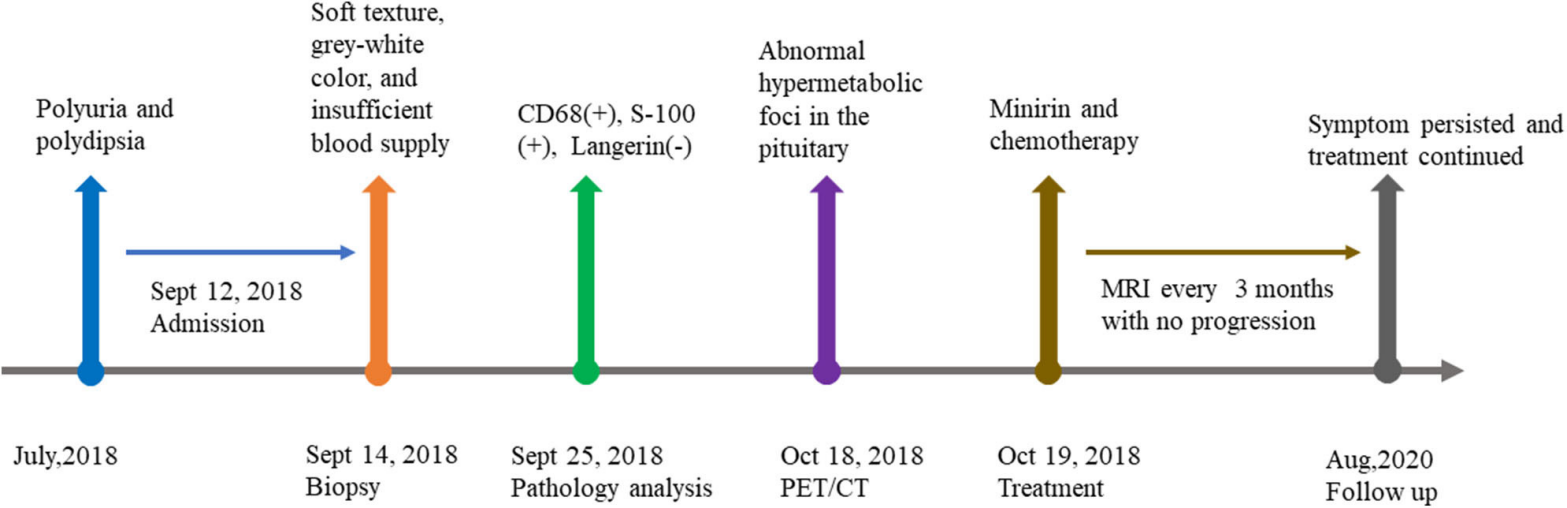
Timeline of the case presentation.

Since histological diagnosis of Rosai-Dorfman disease was confirmed, FDG-PET/CT was performed to explore whether there were systemic lesions. PET/CT revealed abnormal hypermetabolic foci in the pituitary which is considered as residual RDD lesion. Bony changes and hypermetabolic foci were also seen in the right sternal end of clavicle, right transverse process of T10, left acetabulum and right femoral head. No abnormal lesions were found in head, neck, thorax, abdomen, and pelvis (Figure 3).

The patient then started oral minirin treatment (0.25 mg per day) and received oral medication of chemotherapy (mercaptopurine, dose dependent on blood workup) with intermittent corticoid treatment. At the last follow up in August 2020, the patient persisted symptoms of polyuria and polydipsia and continued chemotherapy and minirin treatment. Regular MRI of every 3 months revealed no progression.

## Discussion

RDD is a benign, self-limiting, and progressive lymphoproliferative disorder of unknown etiology and pathogenesis with good outcome (2, 17). It was originally described by Destombes in 1965 (21) and characterized as a distinct clinicopathological disorder in 1969 by Rosai and Dorfman (1). Recently, RDD has been recently classified as part of the “R group” of histiocytoses by the Histiocyte Society in 2016 (22). The prevalence is about 1:200,000 and an estimated 100 new cases/year in the United States. It is more frequently seen in children and young adults (mean age 20.6 years), although it has been reported up to age 74 (5).

We conducted the literature review using PubMed database. Key words of “Rosai-Dorfman disease,” “Rosai-Dorfman disease” with “central nervous system” and “Sinus histiocytosis with massive lymphadenopathy” were used to select for adequate papers up to August 2020 in which the diagnosis of RDD was confirmed histologically. Cases with sellar involvement were included. Only English studies are considered and those articles lacking full text or important information were also filtered out. In addition, we also reviewed references of relevant studies. A total of 14 cases (including our case) were reviewed in terms of clinical presentation, endocrine abnormality, radiological features, pathology, treatment, and follow up.

It has been suggested that dysfunction of the immune system, or an autoimmune process may be the cause of this disease (23). The clinical symptoms vary as a result of the involvement of different organs or systems. The most common neurological manifestations of sellar RDD is diabetes insipidus and visual changes. Others include high cranial pressure, neuropsychiatric manifestations, and cranial nerve paresis, et al. In our analysis, visual disturbance presents in 50% and diabetes insipidus in 42%, followed by headache in 33%. Visual disturbance includes blurred vision, visual field defects and diplopia. Two typical kinds of MRI manifestations in intracranial RDD have been described in our study; one is like meningioma-like mass lesions, another showing infiltrative pattern that demonstrates hyperintense areas on T2-weighted imaging. Heterogeneous contrast enhancement is usually present. These are in agreement with our findings. Fifty-four percentage of the involved lesions are heterogeneously enhanced after Gd injection.

Currently, there is no clear consensus on the treatment strategies. For sellar RDD, surgical treatment is primarily considered and the aim of it is to completely remove or debulk the tumor. Clinical course is determined by the extent and distribution of the lesion. Previous studies showed that the presence of widespread systemic disease appeared to be an ominous prognostic factor (2, 24). In our analysis, 92% of patients underwent operations, including craniotomy and endoscopic surgery. Six patients with sellar RDD achieved complete or partial remission.

Some limitations also should be noted in this study. Firstly, selection bias may be present in this study because severe or unusual cases were more easily to be published. Secondly, Neyman bias gives an inaccurate data analysis. The undiagnosed dead were not included in this study probably because of poor treatment or not performing histological analysis. Thirdly, the relatively smaller number of cases in some studies limited the power to generate statistically significant evidence of disease associations.

In conclusion, we reported a case of sellar RDD and described the largest corpus of case review of RDD with primary sellar involvement. The underlying pathogenesis and origin of sellar RDD are discussed in this article via the analysis of demographic characteristics, clinical presentations, laboratory results, radiological features, pathohistology findings, treatment, and outcomes. We believe the data summarized here will help alert us to this condition and inspire more treatment strategies in the future.

## Data Availability Statement

The original contributions presented in the study are included in the article/supplementary materials, further inquiries can be directed to the corresponding author.

## Ethics Statement

The studies involving human participants were reviewed and approved by PUMCH Institutional Review Board. The patients/participants provided their written informed consent to participate in this study. Written informed consent was obtained from the individual(s) for the publication of any potentially identifiable images or data included in this article.

## Author Contributions

YY, RW, and YZ: conception and design. HP, WM, and YZ: development of methodology. YZ, JL, JZ, XZ, KZ, and SW: acquisition and analysis of data. YZ, JL, and JZ: writing, review, and revision of manuscript. HZ and HP: technical and material support. YY, RW, and HZ: study supervision. All authors have read and approved the manuscript. All authors contributed to the article and approved the submitted version.

## Conflict of Interest

The authors declare that the research was conducted in the absence of any commercial or financial relationships that could be construed as a potential conflict of interest.
